# Enhanced Security Authentication Based on Convolutional-LSTM Networks

**DOI:** 10.3390/s21165379

**Published:** 2021-08-09

**Authors:** Xiaoying Qiu, Xuan Sun, Monson Hayes

**Affiliations:** 1 College of Information Management, Beijing Information Science and Technology University, Beijing 100192, China; sunxuan@bistu.edu.cn; 2 Department of Electrical and Computer Engineering, George Mason University, Fairfax, VA 22030, USA; hayes@gmu.edu

**Keywords:** physical layer security, wireless networks, classification algorithms, deep learning

## Abstract

The performance of classical security authentication models can be severely affected by imperfect channel estimation as well as time-varying communication links. The commonly used approach of statistical decisions for the physical layer authenticator faces significant challenges in a dynamically changing, non-stationary environment. To address this problem, this paper introduces a deep learning-based authentication approach to learn and track the variations of channel characteristics, and thus improving the adaptability and convergence of the physical layer authentication. Specifically, an intelligent detection framework based on a Convolutional-Long Short-Term Memory (Convolutional-LSTM) network is designed to deal with channel differences without knowing the statistical properties of the channel. Both the robustness and the detection performance of the learning authentication scheme are analyzed, and extensive simulations and experiments show that the detection accuracy in time-varying environments is significantly improved.

## 1. Introduction

Innovations in communication technologies and artificial intelligence (AI) over the past two decades have not only brought about tremendous new smart applications, but also significantly increased serious security risks imposed on wireless devices, owing to the openness of radio signal propagation [1,2,3]. An explosive growth in the number of Internet-of-Thing (IoT) terminals provide abundant opportunities for adversaries to intercept transmissions and commit undetected spoofing attacks. In addition, the lack of standardization in security protocols for IoT and intermittent communications is detrimental to the performance of wireless communication systems. Moreover, the complex dynamic network environments and the “on–off” transmissions of resource-constrained devices make it more difficult to authenticate and identify illegal transmissions in wireless networks. Therefore, a proper authentication control mechanism is essential for wireless communication networks, especially considering the continuous integration between the wireless infrastructure and the development of smart industries supported by IoT.

While digital key-based cryptographic schemes are employed for network security, they are based on an assumption that spoofing attackers lack the computational and storage capabilities to successfully attack the network [4]. A fundamental drawback of key-based cryptographic techniques is that a user will either pass a one-time static authentication or fail by a binary security check [5]. Because of the rapid growth in the computing power of smart communication nodes, it is becoming increasingly more likely that a potential intruder will be able to crack privacy keys from received information [4]. Although repeated security checks may be achieved with conventional key-based cryptographic methods by repeatedly logging into the network, this is not a feasible solution since the time delay and computational overhead put a strain on resource-constrained sensor nodes [6,7]. Furthermore, classic authentication protocols based on cryptography primitives rely on proper key management and distribution, which may lead to excessive network traffic delays. These flaws can be found in any wireless security framework and are not necessarily limited to IoT communication systems.

Instead of digital key-based cryptography, an information-theoretic approach may be used, which utilizes the unique characteristics of a wireless medium. These properties are intrinsic to the uniqueness of the wireless device and the corresponding communication environment, which are hard to clone and impersonate for an adversary [8,9,10]. Various physical layer attributes have been considered, including the impulse response of the wideband channel [11], the frequency response of the OFDM transmission [12], and the carrier frequency offset [13]. Although physical layer security has some obvious features, such as low complexity, low computational overhead and low power consumption, most solutions are static and only perform one-time authentication in the time domain [14,15,16,17]. In such a context, they are unsuitable for continuous certification in a dynamically fading environment. Although imperfect estimation and dynamic interference conditions provide unique identification features, they constitute major challenges in authentication. A Gaussian mixture model (GMM)-based learning approach was proposed [18,19] that extracts multiple features to improve the detection accuracy of the security authenticator. However, all of these approaches suffer from the same drawback—they only constitute one-time authentication and rely on the binary static attributes of the wireless medium, and thus have challenges in discovering and tracking the time-varying attributes of legitimate users.

In order to overcome these challenges, a machine-learning approach using an adaptive neural network was proposed to model the security authentication problem [20]. However, this approach, along with many other machine-learning approaches as reviewed in recent survey papers [21,22,23]—approaches such as the SVM methods of [24,25] and the polynomial regression schemes of [26,27]—do not take into account the complex changing channels in real-world scenarios. When the channel estimation samples related to the feature distribution are accurate, these machine-learning schemes are more accurate than traditional methods, but once measurement errors occur in the channel estimation process, these learning-based classifiers have a greater chance of failing [28,29,30,31]. This dramatically limits the viability of using conventional machine-learning techniques in a time-varying fading environment, especially when they use static statistical characteristics for classification. Furthermore, the limited distribution range of the single channel attribute may not be sufficient to distinguish devices all the time. Due to changes in the wireless propagation channels, conventional methods have resulted in physical layer authentication with low reliability and robustness, especially in resource-constrained IoT applications.

Moreover, the authors of [14] investigated physical layer security techniques based on the extreme learning machine algorithm to achieve high detection performance for forged messages. An extreme machine learning-based algorithm is basically a two-layer neural network, where the first layer of the proposed network is trained on random parameters, and the second layer relies on the Moore–Penrose generalized inverse [14]. The authors demonstrated the efficiency and feasibility of the proposed scheme by comparing it with other traditional methods. However, its efficiency critically depends more on the available dataset of channel information estimates. Furthermore, in [18], it is assumed that all channel characteristics obey the same statistical distribution functions, such as the Gaussian distributions; thus, their success is still limited in the non-stationary wireless applications considered in this paper.

Fundamentally, the shift to deep learning has opened up new forms of intelligent endogenous security, due to the learning model’s autonomous nature [32,33,34]. A promising method for modeling the authentication process is learning and tracking physical layer properties using neural networks. One of the advantages of convolutional neural networks is that they can increase the dimensionality of channel features based on multi-layer convolutional mapping [35,36] and model the authentication problem as a nonlinear black box without needing to know the statistical distribution functions of the channel attributes. More importantly, the physical layer characteristics and time-varying fading of non-stationary wireless environments may be tracked by the neural network. All these compelling advantages and features inspire us to consider a new neural network-based authentication approach as an adaptive process in the face of real-world non-stationary channels. By discovering dynamic communication scenarios encountered and tracking changes in a wireless propagation channel, the adaptive authenticator based on deep learning becomes capable of adapting to a dynamically changing wireless network. For improved authentication performance, therefore, it is necessary to deal with time-varying features rather than initial static estimates. Table 1 makes a brief summary of security authentication in a wireless network.

Most of the literature on physical layer authentication using deep learning has not considered the challenge of authentication in time-varying environments. In this paper, this challenge is addressed by designing a Convolutional Long Short-Term Memory (Convolutional-LSTM) network for adapting to continuously changing channel properties that enable the continuous identification of wireless devices. This approach combines feature extraction and classification to achieve intelligent adaptation and seamless overall optimization of the system, thereby reducing the vulnerability of spoofing attacks. Simulations show that this Convolutional-LSTM network has the ability to learn sequential channel data in a changing wireless environment.

Overall, the contributions of this paper are as follows:

- First, a novel framework is proposed that enables the network to verify the reliability of messages and authenticate malicious attackers who seek to degrade the security performance of the system. The proposed approach uses two-dimensional measure information as a security parameter that is used in conjunction with physical layer attributes as the solution to the problem of security authentication against spoofing attackers.
- Second, a detection model is proposed that is based on a Convolutional-LSTM network, which learns dynamic channel features without knowing the statistical distribution function, and reduces the complexity of the authentication process, compared to encryption methods. The resulting physical layer authentication process can be regarded as an intelligent model, which is easier to train, based on the estimation of channel attributes and on the authentication of the results that are observed.
- Third, the performance results from simulations show that the proposed Convolutional-LSTM network model describes an adaptive procedure of security authentication, thereby providing reliable protection for legitimate communication links. The superiority of this authentication process over the conventional learning approaches is demonstrated.

The rest of this paper is organized as follows. In Section 2, the system model used in this paper is presented. In Section 3, the SD-USRP-based dataset for security authentication in wireless environment is introduced. The proposed Convolutional-LSTM network approach is presented in Section 4, and both the convergence as well as our authentication performance analysis are presented in Section 5. Finally, conclusions are presented in Section 6.

## 2. Preliminary

### 2.1. System Model

**Figure 1:**
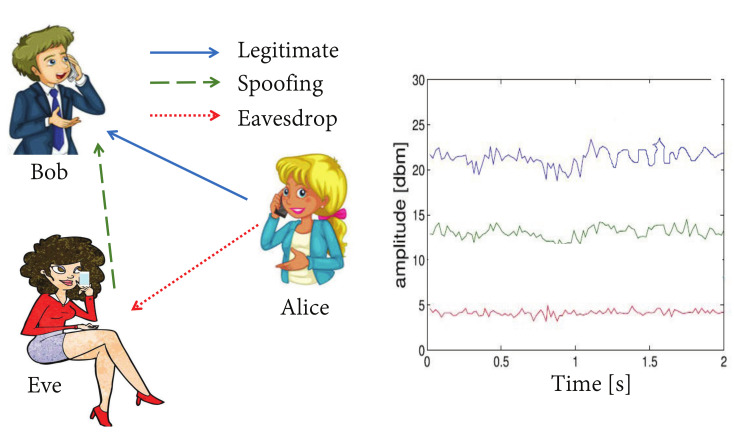
Security authentication system between Alice and Bob based on physical layer characteristics.

As shown in Figure 1, we consider the following channel model in a time-varying communication system, where Alice, Bob and Eve are at geographically different locations. Eve is a spoofing attacker who tries to impersonate a legitimate transmitter, Alice, by sending confused messages to Bob. More specifically, malicious attackers not only attempt to connect to wireless networks, but also try to forge authorized identities in order to obtain illegal access to the authenticator. In a dynamically changing network, multiple copies of the transmitted signal appear through different propagation paths, due to the existence of scattered entities and reflected objects. Therefore, the estimates of the physical attributes between the main channel and the opponent channel are independent and unrelated. As a result, it is difficult for Eve to predict and clone the characteristics of the legitimate wireless channel, such as received signal strength, channel impulse response, and path delay.

It is reasonable to assume that the initial communication transfer between Alice and Bob is established, using the upper-layer protocol before the spoofer arrives, which allows the authenticator to estimate Alice’s physical properties. *M* channel estimations of the legitimate transmitter can be obtained during the initial transmission stage, which are written as follows:

(1) $$H_{A}={\left[H_{A,1},H_{A,2},\cdots ,H_{A,M}\right]}^{T}$$

where each $H_{A,M}$ represents the time-varying vector estimated from transmitter Alice, and *M* is a time index.

The main task of the authenticator, Bob, is to evaluate whether or not the source of a newly estimated message is from Alice by looking at the difference between the vectors $H_{A,1},H_{A,2},\cdots ,H_{A,M}$ and new channel vectors.

The estimated channel vector to be authenticated is denoted by $H_{Q,t}$. Due to the dynamic fading nature of wireless communication environment, the channel characteristics of $H_{Q,t}$ are likely to be dynamic. It is assumed that physical layer authentication starts at time t=1, and the newly estimated vector $H_{Q,t}$ is appended to $H_{A}$, and becomes the ${\left(M+1\right)}^{st}$ channel estimate. Therefore, the physical layer authentication at time t=1 is given by the following:

(2) $$\Delta H_{Q,1}=f\left(H_{A,1},H_{A,2},\cdots ,H_{A,M},H_{Q,1}\right)$$

where f is a function that quantifies the difference between the estimations of wireless channel $H_{A,1}$, $H_{A,2}$, ⋯, $H_{A,M}$ and $H_{Q,1}$. As described in Section 2.2, a two-dimensional measure is used to quantify this difference [19]. If the difference $\Delta H_{Q,t}$ is large, then the signal to be verified is determined to be from the adversary, Eve. It the following, it is assumed that the noise in the channels of both Alice and Eve, which are caused by incomplete channel estimation, measurement error, environmental noise and other factors, are independent and identically distributed [2].

To improve the security performance, a neural network-based authentication approach is used to learn the channel characteristics and detect spoofing attacks in a dynamic fading wireless system. By learning the properties of complex time-varying channels, the reliability and security of the intelligent authentication can be provided for legitimate communications.

### 2.2. Data Preparation and Measure Engineering

In this subsection, we introduce a two-dimensional measure vector (2D-MV) that is the input to the Convolutional-LSTM network. This 2D-MV consists of two components: a Euclidean distance and sample Pearson correlation coefficient [14]. The proposed 2D-MV has the advantage of being relatively easy to compute, being well suited as a similarity measure between two vectors, and working well in averaging out the effects of channel estimation errors [19]. Given the M+1-dimensional channel estimates $\left\{H_{A,1},H_{A,2},\cdots ,H_{A,M},H_{Q,1}\right\}$, the set of Euclidean distances between $H_{A,m}$ and $H_{Q,1}$ are as follows:

(3) $$D_{m,1}=∥H_{A,m}-H_{Q,1}∥$$

and the sample Pearson correlation coefficients at time t=1 are the following:

(4) $$R_{m,1}=\frac{\langle H_{A,m}-\bar{H_{A,m}},H_{Q,1}-\bar{H_{Q,1}}\rangle }{∥H_{A,m}-\bar{H_{A,m}}∥∥H_{Q,1}-\bar{H_{Q,1}}∥}$$

for m=1,2,…,M, where $\bar{H_{A,m}}$ and $\bar{H_{Q,1}}$ represent the means of channel vectors $H_{A,m}$ and $H_{Q,1}$, respectively. Since these measures are imperfectly estimated and time-varying, a multi-dimensional measure space is used to characterize the physical layer properties without knowledge of the distribution functions. More specifically, when a message is uncertain as to whether it is from the attacker or Alice, the following two-dimensional measure vector is created:

(5) $$\Delta H_{Q,1}=f\left(H_{A,1},\cdots ,H_{A,M},H_{Q,1}\right)=\left[\begin{array}{c} D_{1,1},R_{1,1}\\ D_{2,1},R_{2,1}\\ \vdots \\ D_{M,1},R_{M,1} \end{array}\right]$$

After time t=1, this 2D-MV is updated as follows:

(6) $$\Delta H_{Q,t}=\left[\begin{array}{c} D_{1,t},R_{1,t}\\ D_{2,t},R_{2,t}\\ \vdots \\ D_{M,t},R_{M,t} \end{array}\right]$$

The update of $H_{A}$ is done in a way that depends on whether or not the message is from a legitimate transmitter. If $H_{Q,1}$ is from a legitimate transmitter, then it is appended to the $H_{A}$ and the oldest record is discarded:

(7) $$H_{A}={\left[H_{A,2},H_{A,3},\cdots ,H_{A,M},H_{A,M+1}\right]}^{T}$$

On the other hand, if the message is not from a legitimate transmitter, then $H_{Q,1}$ is discarded. In Section 4, we will focus on capturing the local and long-range interdependence of important physical layer features, and enhancing the ability to learn effectively from the time-varying environment.

## 3. Dataset for Security Authentication

To evaluate the performance of the Convolutional-LSTM network-based physical layer authentication scheme that is proposed, we use a Spoofer Detection-Universal Software Radio Peripheral (SD-USRP) dataset that includes two different transceiver pairs in a conference room [20]. The dataset includes legitimate channel estimation and malicious attack data over a period of four days. The USRP transceiver that is used for physical layer attributes acquisition has a center frequency of 2.4 GHz; the wireless protocol is IEEE 802.11 a/g; and the measurement bandwidth is 20 MHz. In our experiments, the sampling frequency of Bob’s receiver is 40 MHz. All channel estimation data in the SD-USRP dataset come from a 6 m × 4 m conference room. Table 2 lists the parameter settings of the experiment.

**Figure 2:**
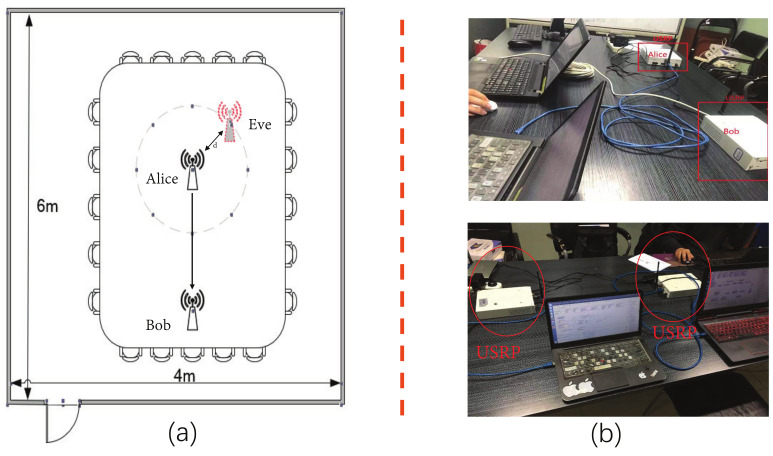
(**a**) Floor plan of the meeting room for data collection. (**b**) Realistic data acquisition scenarios.

Figure 2 shows the experimental setup for the physical layer attribute sampling. The Wi-Fi signal is sent from the sender to the receiving device and is stored in the computer. The legitimate transmitter, Alice, is placed on one side of the table, and the receiver, Bob, is on the opposite side. In the experiment, there was interference between the 802.11 access point near the campus and the client running on the same channel. The attacker, Eve, assumes various positions around Alice. We consider two scenarios: eavesdropping and attacking. In the eavesdropping scenario, Eve eavesdrops on all messages sent from Alice and has no intent to expose herself. In the attacking scenario, Eve may be at any location within a radius of 50 cm away from Alice, and at the same time, sends a malicious message, which allows us to work with the worst case, where Eve has a very similar position (and physical layer attributes) as the legitimate sender Alice. The three locations that are chosen to place the USRP equipment to ensure that the system can effectively classify different transmitters and detect spoofing attacks in the entire area is shown in Figure 2a.

As mentioned previously, since many approaches to authentication, including cryptographic methods and physical layer methods, use one-time authentication and rely on the binary static properties of the wireless medium, the focus here is on non-stationary channels, and addressing the challenges in discovering and tracking the time-varying attributes of legitimate users. Therefore, data are collected, and the dynamic properties are tracked over several days. The dataset consists of 6000×256 samples, with 4000 coming from legitimate communication and 2000 from a spoofing attack. In our experiments, we randomly sample 256 points per frame in the channel estimation process.

## 4. Convolutional-LSTM Network Based Security Authentication

**Figure 3:**
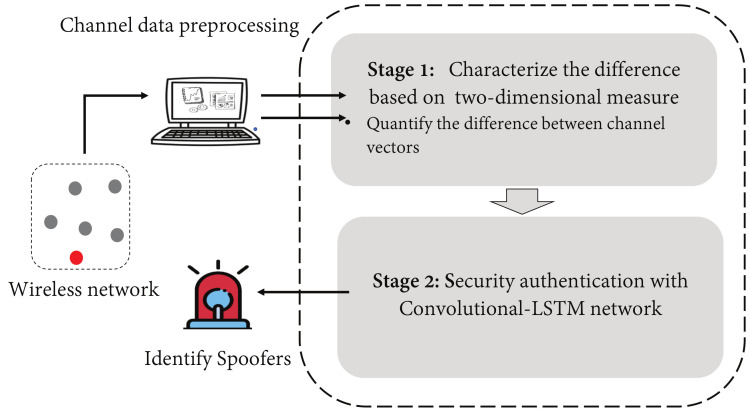
The architecture of the intelligent authentication process.

In this section, the overall architecture of the physical layer authentication scheme that is based on a Convolutional-LSTM network is presented. The overall system is shown in Figure 3. The first step is to quantify the difference between the legitimate channel vectors $H_{A,1},H_{A,2},\cdots ,H_{A,M}$ and a new incoming channel vector $H_{Q,t}$. Then, a Convolutional-LSTM network is used to learn and to track the time-varying physical layer attributes. To classify the new sample $H_{Q,t}$, and to make a determination on whether or not a spoofing attack has occurred, an adaptive authentication framework is used that is based on this two-stage learning system. The advantage of this approach is that it provides continuous and reliable protection by regularly training the online model, thus ensuring that our authenticator can adapt to changing non-stationary channels.

**Figure 4:**
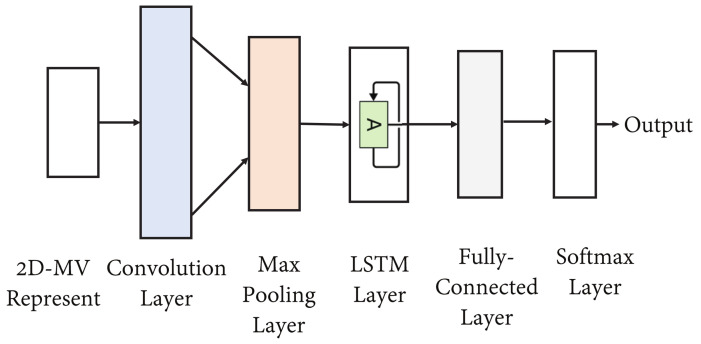
An overview of the intelligent authentication process.

In order to adaptively learn deep features with high discriminative power and optimize the deep classifier, a Convolutional-LSTM network is used to identify the newly estimated physical layer attributes, i.e., $H_{Q,t},t=1,2,3,\ldots$, in a time-varying environment. The architecture is shown in Figure 4.

Generally, physical layer attributes have patterns that change as a result of a time-varying channel. The dynamic characteristics that do not appear in a single channel vector can be dispersed into multiple data vectors. Existing machine-learning approaches for physical layer security authentication fail to track such attributes, and do not have the ability to extract time-varying characteristics that appear in multiple channel vectors. For improved authentication performance, it is necessary to deal with time-varying features rather than initial static estimates. Therefore, after extracting the 2D-MV from original channel signals, the Convolutional-LSTM network is used to simultaneously learn deep features and detect spoofing attackers. Since the 2D-MV $\Delta H_{Q_{t}}$ given in (6) only measures differences and correlations between $H_{A,m}$ and $H_{Q,t}$ over time, the task of the Convolutional-LSTM network is to find a mapping function from the 2D-MV data to features that are able to accurately detect a spoofer.

The convolutional neural network proposed in [20] for physical layer authentication projects estimates of the physical layer attributes into the deep feature space and was shown to have an excellent learning capability. However, due to the vanishing gradient problem, this network is unable to detect long-range interdependencies. Therefore, to learn local characteristics as well as global features, an LSTM layer is inserted into the network. This layer has three gates (input, forget and output) [40]. The cell states ${\mathrm{CS}}_{t}$ in the LSTM module are given by the following:

(8) $${\mathrm{CS}}_{t}={\mathrm{Forg}}_{t}⊙{\mathrm{CS}}_{t-1}+{\mathrm{In}}_{t}⊙\tanh\left(U\cdot {\mathrm{Hid}}_{t-1}+Wx_{t}+b\right)$$

where *t* represents time, ${\mathrm{Forg}}_{t}$ is a forget gate, ${\mathrm{CS}}_{t-1}$ is the memory cell state at time t−1, ${\mathrm{Hid}}_{t-1}$ is a hidden state, $x_{t}$ is the input, *U*, *W* and *b* are network parameters, and ⊙ is the element-wise multiplication operation. The hidden states ${\mathrm{Hid}}_{t}$ are given by the following:

(9) $${\mathrm{Hid}}_{t}={\mathrm{Out}}_{t}⊙\tanh\left({\mathrm{CS}}_{t}\right)$$

**Figure 5:**
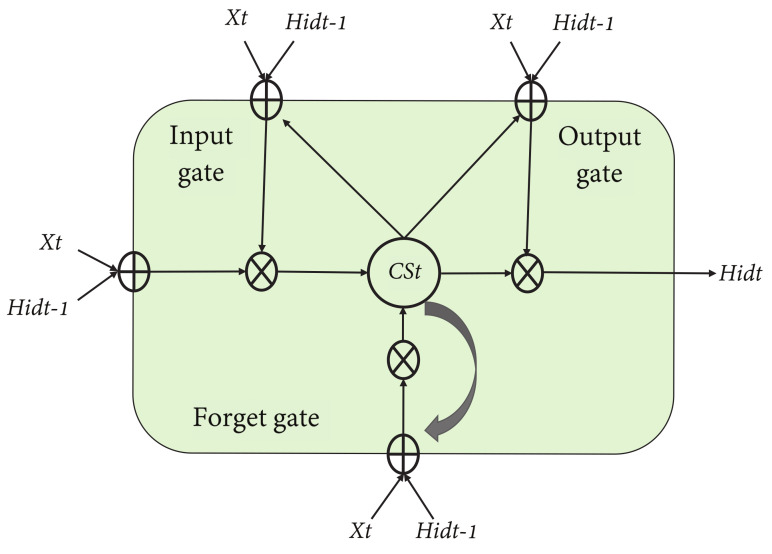
Structure of LSTM layer.

A typical structure of the LSTM layer is illustrated in Figure 5. The input gate ${\mathrm{In}}_{t}$, forget gate ${\mathrm{Forg}}_{t}$ and output gate ${\mathrm{Out}}_{t}$ are related as follows:

(10) $${\mathrm{In}}_{t}=\sigma \left(W_{\mathrm{In}}x_{t}+U_{\mathrm{In}}{\mathrm{Hid}}_{t-1}+b_{\mathrm{In}}\right)$$

(11) $${\mathrm{Forg}}_{t}=\sigma \left(W_{\mathrm{Forg}}x_{t}+U_{\mathrm{Forg}}{\mathrm{Hid}}_{t-1}+b_{\mathrm{Forg}}\right)$$

(12) $${\mathrm{Out}}_{t}=\sigma \left(W_{\mathrm{Out}}x_{t}+U_{\mathrm{Out}}{\mathrm{Hid}}_{t-1}+b_{\mathrm{Out}}\right)$$

where σ denotes the sigmoid function; $W_{\mathrm{In}}$, $W_{\mathrm{Forg}}$ and $W_{\mathrm{Out}}$ are the weights; $U_{\mathrm{In}}$, $U_{\mathrm{Forg}}$ and $U_{\mathrm{Out}}$ are the parameters; and $b_{\mathrm{In}}$, $b_{\mathrm{Forg}}$ and $b_{\mathrm{Out}}$ are the biases. The LSTM layer first determines what previous information should pass through the forget gate ${\mathrm{Forg}}_{t}$. The network then decides what new information should be kept and stored. The proposed LSTM layer is considered a memory unit, which has three gates. Long-term dependencies on physical layer attributes are captured by utilizing the LSTM layer.

In the Convolutional-LSTM network shown in Figure 4, the input data are initially processed by a convolutional layer with a ReLU activation function [41] to extract features from the 2D-MV. This is followed by a max-pooling layer, and the output is sent into the LSTM layer, which models the short-term and long-term physical layer features. The output of the LSTM is input to a fully connected layer with a ReLU activation function [41], which is given by the following:

(13) yReLU=max(0,yTen)

where $y^{Ten}$ denotes an output tensor. The fully connected layer has two neurons, representing the legitimate transmitter Alice and the spoofing attacker Eve. Finally, a softmax layer is used to calculate the probabilities of different target classes. For example, the probabilities of Alice and Eve are respectively calculated by the following:

(14) $$P_{\mathrm{Alice}}=\frac{e^{V_{1}}}{\sum_{class^{\prime }=1}^{2}e^{V_{class^{\prime }}}}$$

(15) $$P_{\mathrm{Eve}}=\frac{e^{V_{2}}}{\sum_{class^{\prime }=1}^{2}e^{V_{class^{\prime }}}}$$

where $V_{1}$ and $V_{2}$ stand for the output of the fully connected layer. Then, we have the predicted value, which can be expressed as follows:

(16) $${\mathrm{label}}_{\mathrm{Pre}}={\mathrm{label}}_{\max(P_{\mathrm{Alice}},P_{\mathrm{Eve}}})$$

The decision of the Convolutional-LSTM network is made by the following:

(17) $$A_{\mathrm{label}}=\mathrm{Eq}\left({\mathrm{label}}_{\mathrm{Pre}},{\mathrm{label}}_{\mathrm{truth}}\right)$$

where Eq is set to one, if ${\mathrm{label}}_{\mathrm{Pre}}={\mathrm{label}}_{\mathrm{truth}}$, otherwise Eq is set to zero. The whole network is trained and optimized through backpropagation with a loss function given by the following:

(18) $$L=-\sum_{class=1}^{N}y^{class}\log\frac{e^{V_{class}}}{\sum_{class^{\prime }=1}^{N}e^{V_{class^{\prime }}}}$$

where *N* is the number of target classes, *V* is the output vector of the fully connected layer in the network model, and $y^{class}$ represents a label vector. There is only one truth label whose corresponding $y^{class}$ value is set to one, and all other $y^{class}$ values are set to zero.

During training, each channel vector (2D-MV) is input to the network, and deep features are generated by the convolution and LSTM layers. The bias between the truth label and the prediction is calculated by (18). The Convolutional-LSTM network uses two kinds of tags corresponding to two types of channel vectors for training. Notably, there may be multiple spoofing attackers, and multiple classifications may be of interest. Often, however, it is of interest only to classify channel vectors as legitimate or illegitimate. All physical layer attributes that do not belong to Alice are considered to come from malicious attackers. In this case, the Convolutional-LSTM network is trained to perform binary class authentication. In this case, the dataset is split into a training dataset (80%) and a test dataset (20%). At the same time, the 20% samples in the training dataset are used for verification.

During testing, the test dataset is fed into the proposed Convolutional-LSTM network, and the probability of the channel vectors belonging to different transmitters is estimated. More specifically, the prediction used in the model can be calculated as (14) and (15). The overall authentication scheme is summarized in Algorithm 1.
**Algorithm 1** Security authentication based on Convolutional-LSTM network.Given physical layer attribute used H, Alice’s estimates $H_{A,m}$, m=1,2,…,M, initialize the network parameters1:**Iteration**:2:      Generate training dataset;3:      Obtain *D* and *R* via (3) and (4);4:      Calculate the 2D-MV ΔH via (2) and (5);5:      Input 2D-MV into the network;6:      Calculate the loss *L* via (18);7:      Update the weight *W*;8:      Adjust the neural network through (8)–(12);9:      Obtain the trained model;10:**Adaptive authenticator**:11:      Receive a new channel vector $H_{Q,t}$12:      Obtain predicted result using Pr via (14) and (15);13:      **if** the channel vector $H_{Q,t}$ is from Alice **then**14:            Accept the message;15:            Update the input dataset via (1) and (6);16:      **else**17:            Terminate communication;18:      t=t+1;

## 5. Experimental Results

In this section, the experimental results are presented that show the effectiveness of the authentication process using the Convolutional-LSTM network. The section begins with a description of the setup used to evaluate the network. In the experimental results section, a comparison between this network and other approaches is given, and its superiority over a non-deep learning benchmark is demonstrated.

### 5.1. Experimental Setup

The Convolutional-LSTM is trained using the 2D-MV, which is extracted from the channel estimates. One physical layer attribute is considered, namely, the received signal strength $H_{RSS}$, in order to verify the viability of the Convolutional-LSTM network. The received signal strength can be written as $P_{loss}=75+36.1\log\left(d/10\right)$, where $P_{loss}$ represents the path loss, and *d* denotes the distance between the transceivers. The total number of network layers is 6, and the initial parameters of the network are random. The hyperparameters of the network are given in Table 3. After training, the network is evaluated in an indoor conference room to evaluate its performance.

Once the Convolutional-LSTM network is trained, it is evaluated on the test set, and the authentication performance is measured in terms of its detection accuracy and false alarm rate, which are the following:

(19) Accuracy=Pr(Alice|Alice∪Eve)

(20) Falsealarm=Pr(Eve|Alice)

These are the most basic parameters for the physical layer authentication performance evaluation.

### 5.2. Impact of 2D-MV Using Measurement Data

**Figure 6:**
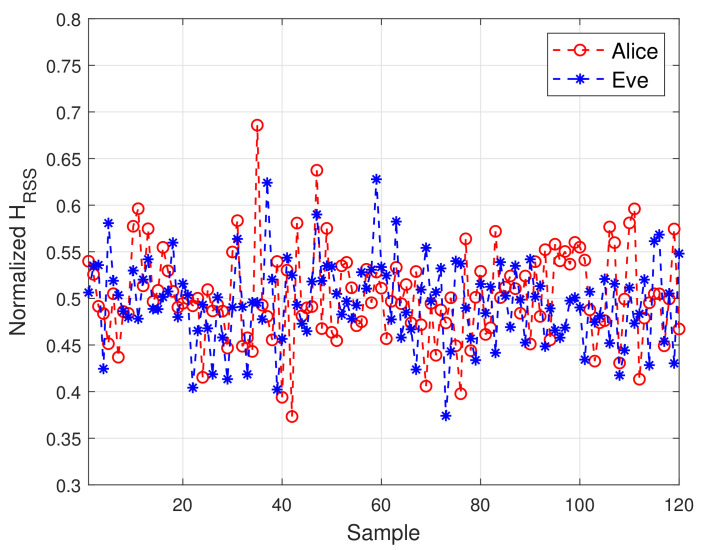
Normalized channel signal estimates of Alice and Eve.

Given the parameter sets in Table 3, the physical layer attribute estimates of different transmitters can be obtained in the indoor meeting room where their received signal strength of (1) is shown in Figure 6. In the intelligent process we proposed, the channel feature space 2D-MV should be determined, namely, for the Euclidean distance *D* and Pearson correlation coefficient *R*, corresponding to the received signal strength. Figure 6 characterizes the estimated channel vector $H_{A,m}$ and the preprocessed signal through stage 1. The channel vectors are updated for each estimation.

**Figure 7:**
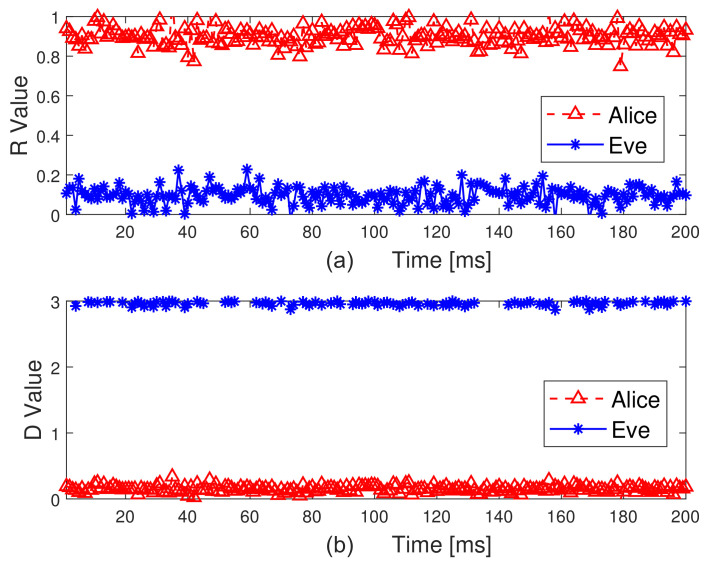
The feature distribution of different scenarios. (**a**) Sample Pearson correlation coefficient. (**b**) Euclidean distance.

**Figure 8:**
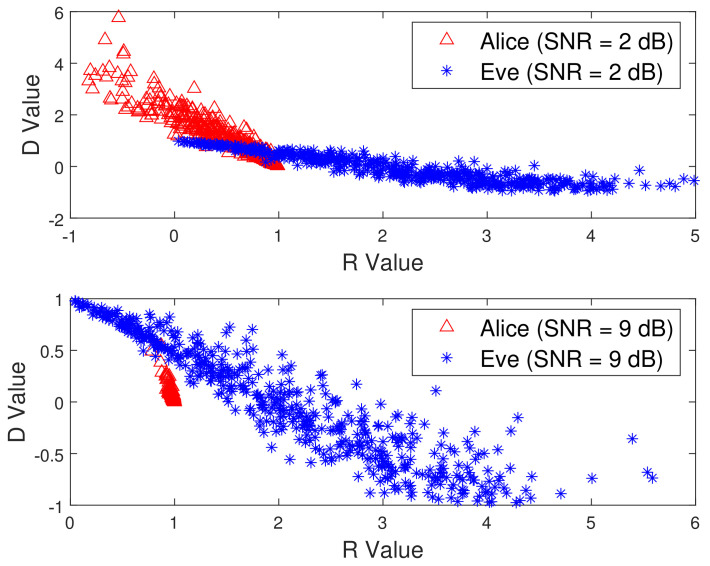
Characteristic distribution under different SNRs.

To test the effectiveness of the proposed scheme, Figure 7 plots the sample Pearson correlation coefficient and Euclidean distance, i.e., D and R, for 200 ms of measurements. In Figure 7, there is a clear gap between the data corresponding to the presence and absence of Eve. This is because 2D-MV not only removes noise, but also eliminates some interference caused by channel estimation errors. Although the combination of these two features improves the difference between Alice and Bob, the result of security authentication still suffers from unsatisfactory performance due to the complex non-stationary environment. The measured vectors under different communication conditions are shown in Figure 8. This is expected, as the non-adaptive classifiers will always have prediction error, even when the history $H_{RSS}$ is known perfectly. We can observe from Figure 7 and Figure 8 that the use of 2D-MV makes our approach have a more obvious improvement in the feature space distribution. Therefore, we chose 2D-MV as the input of the Convolutional-LSTM network for malicious attack detection.

### 5.3. Convergence Performance

**Figure 9:**
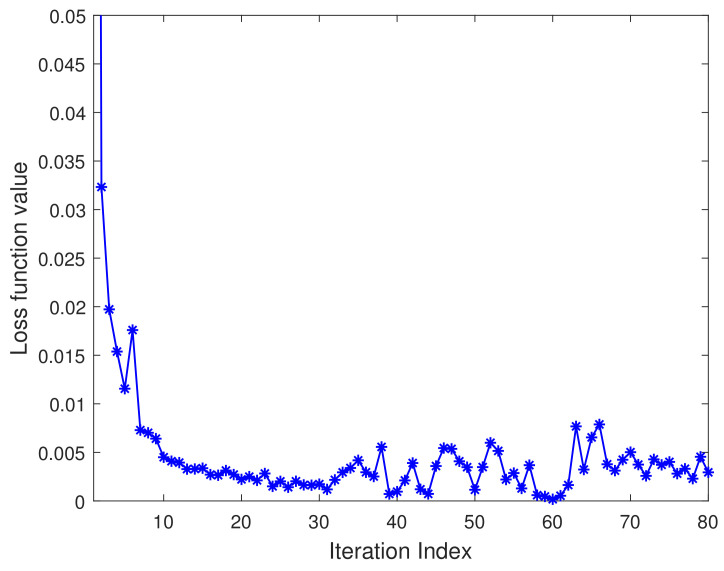
Convergence performance of the Convolutional-LSTM network.

Figure 9 characterizes the convergence performance of the Convolutional-LSTM network (see Algorithm 1), using the 2D-MV feature vectors as the inputs to the network. We consider the use of the received signal strength to authenticate malicious attackers. We can observe from Figure 9 that, with the increasing iteration index, the loss values of the detection scheme dramatically decrease. The reason for this trend is that the Convolutional-LSTM network is an authentication algorithm that can update the system according to the dynamic characteristics of wireless channels so as to adapt to the changing non-stationary environment. Furthermore, the combination of the Euclidean distance and sample Pearson correlation coefficient in the 2D-MV-based feature space can reduce the impact of imperfect channel estimation on authentication performance.

### 5.4. Impact of the Iteration Index and the SNR

**Figure 10:**
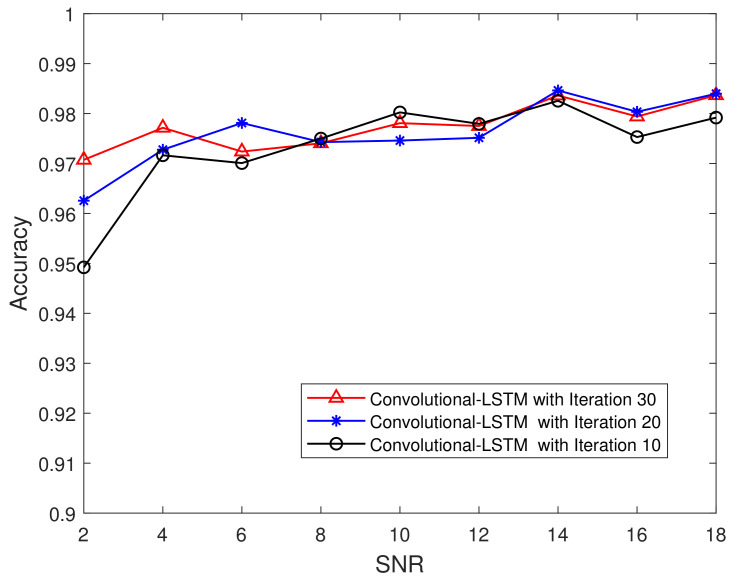
Performance comparison—SNR changes from 2 dB to 18 dB.

Figure 10 compares the performance of the Convolutional-LSTM network when the SNR changes. To account for complex scenarios, we added a sequence of Gaussian white noise, which caused the SNR to change from 20 dB to 2 dB. From Figure 10, we can see the similar trends as in Figure 8. The advantage of the Convolutional-LSTM network is that it does not require the statistical characteristics of the channel, and intelligent training can make the model adapt to changes in the environment.

### 5.5. Authentication Accuracy Performance

**Figure 11:**
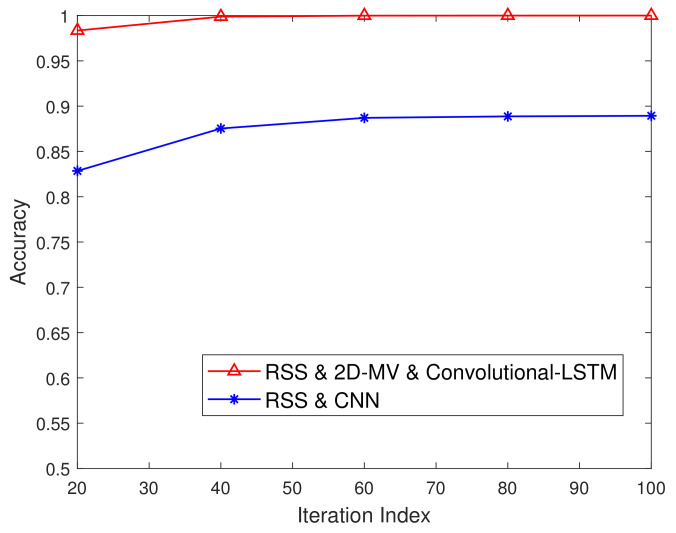
Authentication performance of the Convolutional-LSTM network and the CNN-based approach of [20].

Figure 11 characterizes the detection accuracy vs. the iteration index. As we discussed before, the proposed security authentication scheme related to the classifier gradually converges to a steady-state value after 20 iterations. In Figure 11, we describe the detection performance of the learning-based authentication scheme. It can be observed from Figure 11 that the detection accuracy of the Convolutional-LSTM network proposed in this paper is better than that of the authentication process based on the convolutional neural network. The utilization of 2D-MV for characterizing the difference is extremely helpful to provide high protection for legitimate users.

**Figure 12:**
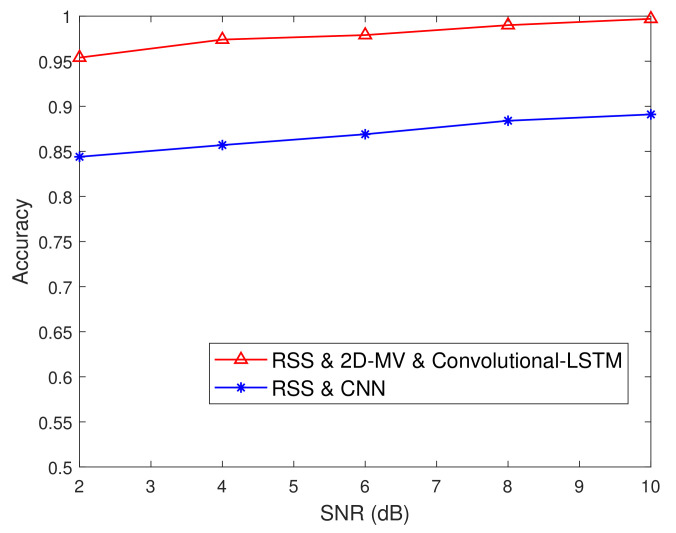
Comparison of results of the Convolutional-LSTM network and the CNN-based approach of [20] in a conference room scenario.

Figure 12 characterizes the comparison results of our proposed physical layer authentication approach and the method based on a convolutional neural network [20]. It can be seen from Figure 12 that our adaptive framework performs much better than the convolutional neural network-based approach. Furthermore, the existing method of using static channel characteristics also limits its application in dynamic wireless networks. More importantly, the Convolutional-LSTM network has shown obvious advantages in the process of learning local and global features, while the traditional machine learning methods cannot provide such dynamic features during the authentication process (please see Figure 1).

### 5.6. Evaluation of the Convolutional-LSTM Network System

Table 4 shows the results of different detection classifiers at each stage. We try to change the type of classifier to observe the accuracy of the authentication approach. It can be observed that the classic SVM-based physical layer authentication performs quite poorly, with a detection accuracy of only 68%. The GMM-based authenticator manages to significantly improve the accuracy, showing a detection rate of up to 89%. However, the most significant improvement we experienced is the use of the Convolutional-LSTM network, which successfully detected malicious attackers with a recognition accuracy of up to 99%. The advantages of CNN in deep feature learning and the long-term dependence between non-linear features are the main factors leading to this significant improvement in the performance of this authentication. More importantly, compared with other classifiers we used, the Convolutional-LSTM network is an intelligent model, which can learn time-varying information between different channel vectors. The computational complexity of the SVM-based authentication approach is relatively small. However, the detection accuracy of SVM is only 68.35%, which is much lower than 99.15% of Convolutional-LSTM. More weight parameters in the Convolutional-LSTM network need to be trained. However, the dimension of the input (2D-MV) in the Convolutional-LSTM-based algorithm is much smaller than the authentication approach based on CNN.

In Table 5, we can observe the comparison of results between our proposed solution and the existing methods, using the SD-USRP dataset. According to the available evaluation results of the compared schemes, we selected the best results for each authentication method in terms of accuracy and false alarm rate. We can observe that our proposed authentication system performs better both in relation to the accuracy and the false alarm rate. This is mainly because we have adopted an efficient measure space technique and proposed a suitable Convolutional-LSTM network. It is worth noting that these parameters are for reference only because many researchers use different datasets and preprocessing and sampling techniques. Therefore, it is usually not appropriate to directly compare some metrics (such as training and testing time), although our solution has made improvements in all performance indicators and performed better than other methods. Nevertheless, we point out that using the proposed authentication approach can achieve a remarkable level of security against spoofing attackers that has high robustness, strong resistance to surrounding interference and adaptive updates. In summary, we have summarized the authentication methods used along with their advantages and costs in the taxonomy Table 6.

## 6. Conclusions

In this paper, we proposed an adaptive physical layer authentication scheme to improve the authentication performance from the perspective of robustness and reliability. The Convolutional-LSTM network was designed to model the authenticator as an intelligent learning system, which effectively alleviates the imperfectness of the channel attribute estimation. By proposing a multi-measurement space, i.e., the combination of Euclidean distance and sample Pearson correlation, the dimension of the feature-space was increased under the time-varying channel attributes. Both the convergence performance and authentication performance of our Convolutional-LSTM network solution were analyzed and verified in a realistic indoor conference room. The experimental results show that the proposed security authentication framework has better detection performance than some existing methods, such as the non-adaptive approach and the convolutional neural network-based counterpart.

## Figures and Tables

**Table 1:** Different security authentication paradigms.

Authors	Contributions and Concepts
B. Chen et al. [37] P. Moulin et al. [38] A. Zaidi et al. [39]	Information hiding strategies in the presence of attackers are proposed for partial or non-cooperative mode.
N. Wang et al. [14]	A physical layer authentication based on extreme learning machine algorithm is proposed which does not require key generation.
X. Qiu et al. [18]	An authentication model based on Guassian mixture model using static characteristic is proposed,
R. Liao et al. [35]	A multi-layer convolutional mapping method without needing to know the statistical distribution functions of the channel attributes is proposed
Our paper	A deep-learning method for physical layer authentication based on continuous characteristics is proposed

**Table 2:** The parameter settings.

Parameters	Values
Carrier Frequency $f_{c}$	2.4 GHz
Channel Model	Rayleigh Fading Model
Number of Subcarriers	256
Wireless Protocol	IEEE 802.11a/g
Signal to Noise Ratio (SNR)	2 dB∼20 dB
Bandwidth	20 MHz
Sampling Interval	2.5 ms
Distance between Alice and Bob	1 m

**Table 3:** The training hyperparameters.

Settings	Values
Initialization Parameters of Model	random
Number of Layers	6
Learning Rate	5$\times 10^{-3}$ (20)
Training Subsets	4000 × 256
Validation Subsets	800 × 256
Testing Subsets	1200 × 256
Batch Size	128
Number of Epochs	30

**Table 4:** Detection performance using different classifiers.

Classifier	Accuracy	False Alarm Rate	Stage
SVM	68.35%	15.27%	1
GMM	89.12%	8.13%	1
CNN	95.81%	5.72%	2
Convolutional-LSTM	99.15%	0.71%	2

**Table 5:** Detection performance in different schemes.

Reference	Approach	False Alarm Rate	Accuracy
Scheme [19]	PCA + GMM	7.92%	94.50%
Scheme [18]	KLT + GMM	7.72%	92.80%
Scheme [20]	CNN	5.72%	95.81%
Scheme [36]	RNN	1.20%	97.40%
Our scheme	Convolutional-LSTM	0.71%	99.15%

**Table 6:** Taxonomy of detecting malicious attacker use cases.

Algorithm	Accuracy	Complexity	Training Time	Benefit	Cost
SVM	Fair	Low	Low	Require low computational resources	Lack of large-scale deployment
GMM	Fair	Fair	Low	Adaptive Learning	Lack of realistic authentication tests
RNN	High (97.40%)	High	Fair	Accurate Learning	Complex learning implementation
CNN	Fair	Fair	Low	Robust learning	Sensitive to channel variance
Proposed Method	High (99.15%)	Fair	Fair	Improved learning efficiency; real experiments	Training privacy

## Data Availability

Not applicable.

## Abbreviations

The following abbreviations are used in this manuscript:

GMM	Gaussian mixture model
IoT	Internet of Things
MHz	Mega Hertz
RSS	Received signal strength
LSTM	Long short-term memory
2D-MV	Two-dimensional measure vector
USRP	Universal software radio peripheral
SVM	Support vector machine
CNN	Convolutional neural network
RNN	Recurrent neural network
PCA	Principal component analysis
